# Impact of daily auditing and weekly feedback on process of care and patient outcome in resuscitation of severe sepsis and septic shock

**DOI:** 10.1186/cc11703

**Published:** 2012-11-14

**Authors:** B Afessa, CB Bowron, RD Danielson, K Ramar

**Affiliations:** 1 Mayo Clinic, Rochester, MN, USA

## Background

Rivers' randomized clinical trial showed that early goal-directed therapy improves patient outcome in severe sepsis and septic shock [1]. The findings were confirmed by multicenter international prospective observational studies [2,3]. In our institution, we implemented a Quality Improvement Project based on daily auditing of patients admitted to a medical intensive care unit (MICU) for severe sepsis/septic shock followed by weekly feedback on compliance of the Institute for Healthcare Improvement (IHI) resuscitation bundle, and Sepsis Response Team (SRT) activation [4]. Once the Quality Improvement Project was completed, the daily audit and weekly feedback were stopped. This study aims to assess the impact of this change on the process of care and hospital mortality.

## Methods

The study was conducted in a 24-bed adult MICU of an academic medical center. Only the first admission of each patient was included. Patients who did not authorize their medical records to be reviewed for research were excluded. During the first period of the study, 2009, daily auditing, weekly feedback to healthcare providers and SRT were implemented. During the second study period, 2010, the daily auditing and weekly feedback were stopped and the SRT activation was continued. Baseline data collected during the two periods of the study included gender, age, and resuscitation preference in case of cardiac arrest. Process of care was measured using the seven elements of the IHI resuscitation bundle. Patient outcome was measured by hospital mortality. Statistical comparisons between the two groups were made using Student's *t *and chi-square tests. Two-tailed *P *< 0.05 was considered statistically significant.

## Results

A total of 777 patients were included in the study: 378 had auditing and feedback and 399 did not. The mean (SD) age of the feedback group was 65.4 (16.77) years compared with 66.3 (16.67) years for the nonfeedback group (*P *= 0.459). Female gender accounted for 147 (38.9%) of the feedback group compared with 206 (51.6%) of the nonfeedback group (*P *< 0.001). At MICU admission 315 (83.3%) of patients in the feedback group preferred full resuscitation in case of cardiac arrest compared with 317 (79.4%) in the nonfeedback group (*P *= 0.165). Compliance with all elements of the bundle declined from 50.8% during feedback to 29.8% during nonfeedback (Table 1). However, the hospital mortality rate did not increase.

**Table 1:** Process of care and patient outcome differences between the two groups

Variable	Feedback (*n *= 378)	No feedback (*n *= 399)	*P *value
Lactate measured	369 (97.6%)	376 (94.2%)	0.018
Blood culture	364 (96.3%)	370 (92.7%)	0.030
Antibiotic	344 (91.0%)	342 (85.7%)	0.022
Fluid achieved CVP	281 (74.3%)	238 (59.6%)	< 0.001
Vasopressor	331 (87.6%)	330 (82.7%)	0.057
RBC transfusion	331 (87.6%)	336 (84.2%)	0.180
Inotrope	220 (58.2%)	146 (36.6%)	< 0.001
Compliance with all	192 (50.8%)	119 (29.8%)	< 0.001
Hospital death	92 (24.3%)	73 (18.3%)	0.040

## Conclusion

Continuous auditing and feedback to healthcare providers is an important component of a Quality Improvement Project to maintain compliance with the severe sepsis resuscitation bundle. However, it is not associated with hospital mortality.
